# Merging logical models: An application in Acute Myeloid Leukemia modeling

**DOI:** 10.1101/2024.09.13.612961

**Published:** 2024-09-19

**Authors:** Luna Xingyu Li, Boris Aguilar, John H Gennari, Guangrong Qin

**Affiliations:** 1 Institute for Systems Biology, Seattle, WA 98109, United States of America; 2 Department of Biomedical Informatics and Medical Education, University of Washington, Seattle, WA 98195, United States of America

**Keywords:** gene regulatory networks, logical models, model integration, acute myeloid leukemia, systems biology

## Abstract

Gene regulatory network (GRNs) models provide mechanistic understanding of the gene regulations and interactions that control various aspects of cellular behaviors. While researchers have constructed GRNs to model specific sets of gene regulations or interactions, little work has been made to integrate or merge these models into larger, more comprehensive ones that could encompass more genes, and improve the accuracy of predicting biological processes. Here, we present a workflow for merging logical GRN models, which requires sequential steps including model standardization, reproducing, merging and evaluations, and demonstrate its application in acute myeloid leukemia (AML) study. We demonstrate the feasibility and benefits of model merging by integrating two pairs of published models. Our integrated models were able to retain similar accuracy of the original publications, while increasing the coverage and explainability of the biological system. This approach highlights the integration of logical models in advancing system biology and enhancing the understanding of complex diseases.

## Author summary

In our study, we tackle the challenges of integrating gene regulatory network (GRN) models to enhance our understanding of complex biological systems. GRNs are essential tools for understanding how genes regulate various cellular behaviors, but individual models often focus on specific sets of genes or interactions. We present a novel workflow that merges these individual logical GRN models into more comprehensive ones, providing a broader view of gene regulation. We applied this workflow to Acute Myeloid Leukemia (AML), a highly aggressive form of blood cancer. AML is challenging to treat due to its genetic complexity and the frequent occurrence of treatment-resistant mutations. Our integrated models retain the accuracy of the original models while offering improved coverage of the biological processes. This approach offers valuable insights into the disease’s underlying mechanisms through a combination of models that describe different aspects of AML. We envision that the proposed workflow will improve predictions, generate deeper insights, and improve our understanding and treatment of complex diseases like AML.

## Introduction

Gene regulatory networks (GRN) play important roles in a wide range of biological processes, including cell differentiation, signaling transduction and the cell cycle (1). It involves molecular regulators and effectors that interact in complex ways to regulate diverse processes. The dynamics of the GRN are modeled in different ways, including logical models, ordinary differential equation (ODE) models, and piecewise linear differential equation models (2). Among them, logical models are arguably the simplest. This is partly due to limited information on kinetic parameters besides network structure, but primarily because logical models are intuitive and versatile (3,4).

Logical GRN models are typically represented as directed graphs, where nodes represent genes or proteins, and edges represent regulatory interactions. Each node can be in one of several states, which indicate its activation status. For Boolean models, states are binary, meaning a gene or protein can be either active or inactive; and for multi-value logical models, states can have multiple levels of activity. Logical functions used to describe the regulatory interactions consist of a set of logic gates (AND, OR, NOT) (5). Compared with interaction graphs, they capture not only pairwise relationships, but also interactions between more than two components, which is common in biochemical reactions and signaling transductions (6). Since Kauffman’s study in 1969 (7) logical models have been used to describe a wide range of activities in biological systems, from gene activities and protein expression to cellular behavior and system-level phenotypes, ultimately contributing to clinical and translational medicine (For reviews, see (2,4,8,9)).

The construction of logical GRN models requires domain expertise for specific processes. Due to the limitation of knowledge from individual research teams, and the focus of different studies, existing published logical models are mainly focused on one process or theme, with limited coverage of genes and processes for a more systematic study. Different models also used different representations for the nodes and rules, which requires a more systematic approach to merge these models in order to gain more explainability and predictive power. A method to systematically integrate previously developed GRNs can provide more comprehensive models to get more insights on the structured organization and frequent interactions of biological entities. Such integrated models are essential for elucidating complex biological phenomena and understanding the intricate regulatory mechanisms within cells (10,11).

Logical GRN models have been applied to study various diseases, including breast cancer (12), pancreatic cancer (13), acute myeloid leukemia (AML) (14). In this study, we focus on modeling AML, an aggressive hematopoietic malignancy characterized by the accumulation of somatic genetic changes that disrupt normal cell maturation, proliferation, and differentiation of hematopoietic progenitor cells (15–20). AML cells from different patients often carry mutations in genes such as *FLT3*, *DNMT3A*, *NPM1*, *WT1*, *NRAS*, *RUNX1*, *TP53*, and *CEBPA* (17,18,20,21). Patients with different mutations are also associated with different drug responses (20). These mutations are associated with functional alterations in various signaling pathways involving both the mutated genes themselves and their downstream targets. Numerous GRN models have been developed to explore different aspects of AML, including the regulation of hematopoietic stem cells (HSCs) (22), potential drug responses (23) and predictions of clinical outcomes (14). Merging these models offers a promising approach to creating a more comprehensive GRN model for AML. Such an integrated model can better represent the complexity of the disease, providing a more accurate system for simulations, improving drug response predictions, and helping to identify patient-specific therapeutic targets.

In this study, we developed a workflow for merging logical GRNs and applied it to integrate two pairs of published AML models. By combining models that share common components and high biological relevance, we created larger, more comprehensive models that replicate the behavior of individual models while offering broader insights. These merged models are better suited for predicting gene expression and clinical outcomes in AML patients. The workflow includes key stages such as model identification, composition, and evaluation of the merged models. Through this process, we demonstrate the effectiveness of our framework in enhancing the predictive power and understanding of GRNs in AML. Moreover, this workflow is also adaptable for application in other disease models.

## Results

Starting with an overview of the workflow, we next apply it to AML studies and present our findings across several key stages of the workflow: literature search, model identification, model merging, and the evaluation of the merged models. Through this process, we demonstrate the utility of our workflow in enhancing the understanding and predictive power of gene regulatory networks in the context of AML.

### Overview of the model merging workflow

**Fig 1:**
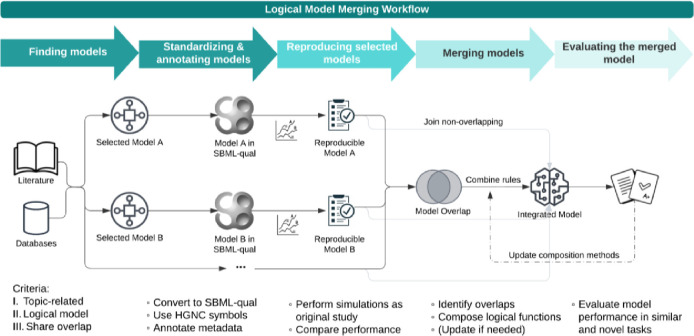
Overview of the logical model workflow. This flowchart depicts the step-by-step process for merging logical models. The workflow starts with the collection of eligible models from literature and databases, which are then converted to the standardized SBML-qual format with added annotations. Next, the reproducibility of each model is verified before proceeding to the next steps. During merging, overlapping components can be combined using different approaches, while non-overlapping components are directly integrated. The approaches tested here include the ‘AND’, ‘OR’, and ‘Inhibitor Wins’ methods. Finally, the integrated mode is evaluated using both similar tasks as in their original studies and novel tasks of interest.

The model-merging workflow integrates logical models from various sources into a unified model with a more complete representation of biological or pathological mechanisms. The workflow consists of five main steps: (1) Finding models, (2) Standardizing & annotating models, (3) Reproducing selected models, (4) Merging models, and (5) Evaluating the merged model, as shown in Fig 1. For further details on each step, please refer to the Methods section.

Initially, the process involves identifying candidate models by reviewing existing literature, repositories, and databases. The models selected are required to have shared genes in the GRNs. After identifying relevant models, they were standardized and annotated using the SBML-qual format (24,25), with gene names and protein identifiers aligned to international standards like HGNC approved symbols (26). Ensuring reproducibility is a crucial step; we rigorously tested the selected models to verify that they can replicate the behaviors described in their original publications.

Following the preparation of the models, the next step involves merging the models using different logical combination methods. We propose three approaches: the ‘OR’ method, which combines activation scenarios inclusively; the ‘AND’ method, which only activates nodes when all models agree; and the ‘Inhibitor Wins’ method, where inhibitory interactions take precedence. Each method is tailored to capture different aspects of biological regulation, depending on the study’s goals. (See methods section for details.)

Finally, we evaluated the performance of the merged models by comparing their predictive accuracy and robustness against both the original models. We also applied the merged model to new, untested scenarios to test if the model results are aligned with experimental measurements. This evaluation helps identify the most effective integration method, and sets the stage for applying this workflow to AML studies, as discussed in the following sections.

### Selecting AML-related logical models

We conducted a broad literature search to identify relevant publications discussing logical models related to gene regulation in AML. Because AML originates from hematopoietic stem or progenitor cells that have undergone malignant transformation (27), our search included models that describe HSC behavior. Understanding the differentiation pathways of HSCs is crucial for identifying how disruptions in these processes can lead to AML, as many mutations associated with AML affect genes involved in normal HSC differentiation, such as *RUNX1* (28) and *CEBPA* (29).

Initially, 93 papers were retrieved; after review, we identified 19 of them that included a qualitative logical model and that were relevant to AML. Details of all 19 selected models, including model type, purpose of the model and validation information, are summarized in Table S2.

These 19 AML logical models share common purposes and validation approaches. Many rely on a combination of literature reviews, experimental data, and public knowledge resources or databases such as KEGG (30) and SIGNOR (31) for understanding molecular interactions and regulatory networks in AML. Most models use Boolean networks to describe interactions among genes and proteins, with some employing probabilistic Boolean models (32) or multi-valued models (33). Various computational tools, including GINsim (34), MaBoSS (35), BioModelAnalyzer (BMA) (36), Reactome FIViz (37), and caspo (38), are used for model construction and simulations. Validation methods primarily include in silico approaches, where the simulated behavior of the model is compared to previously generated experimental data or literature (used in 13 out of the 19 models). In vitro validation, involving laboratory testing of model predictions, is employed in 3 studies (23,39,40), while in vivo validation was not used in any of the models. Some models focus mainly on theoretical or computational aspects without extensive validation.

An important observation from our review is the lack of standardization and accessibility of these models, which leads to challenges in reproducibility. If model results are not reproducible, that greatly reduces their utility for further research. Our work underscores the need for improved standardization and accessibility, which is crucial for model reuse and model composition (41,42).

**Fig 2:**
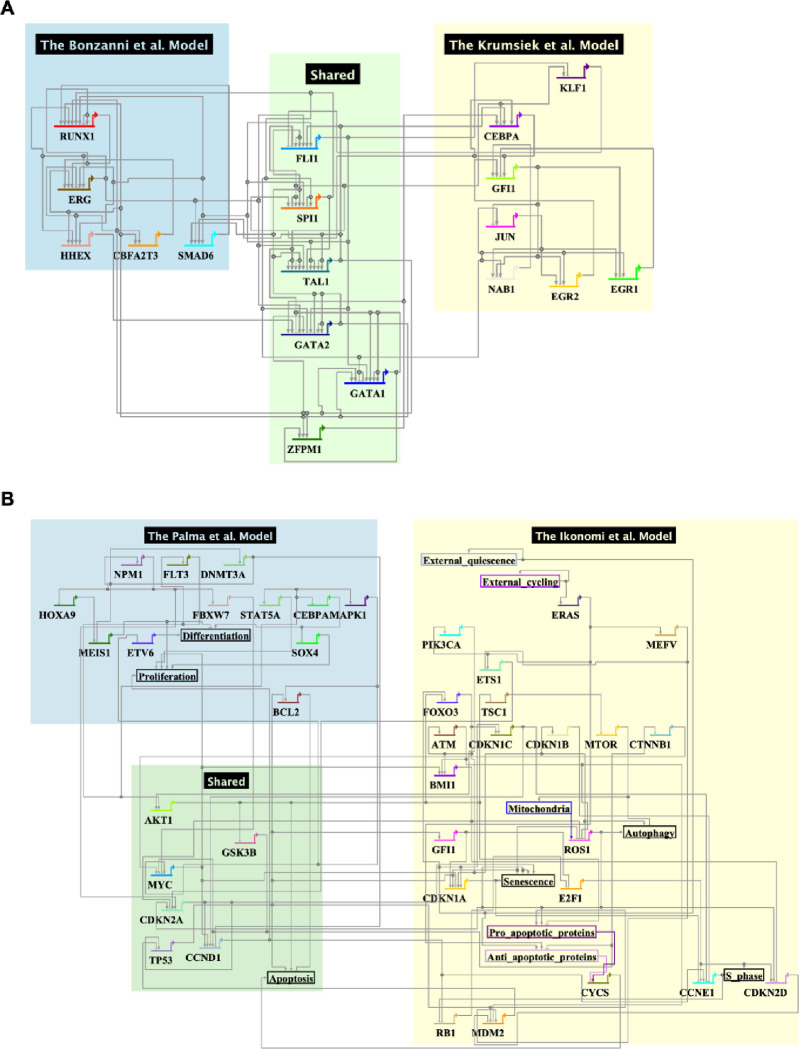
Diagram of the two merged logical models. (A) Merged model using Palma et al. 2021 and Ikonomi et al. 2020; (B) Merged model using Bonzanni et al. 2011 and Krumsiek et al. 2013. Both pairs of models are visualized using BioTapestry (43) with a hierarchical layout. Nodes from the individual models are shaded in blue and yellow respectively, and shared nodes are shaded in green. Each gene is represented as a horizontal line, and other nodes (signals, phenotypes) are framed by boxes. Lines indicate regulation relationships that point from the regulator to its targets, with arrowheads as activating and bars as repressing. Detailed logical rules and SBML-qual file of the models are available in the supplementary material.

From the 19 models, we next selected two pairs of models to demonstrate our workflow for model merging. We searched for pairs of models that share some degree of overlap and where merging is biologically plausible. Table 1 provides overview information about the 4 models, and Fig 2 shows a detailed graphical view of the models, including overlapping genes.

In Fig 2(a), the Bonzanni et al. model (39) captures key regulatory genes of early hematopoietic stem/progenitor cells (HSPCs) and simulates the differentiation of stem cells into mature blood cells, including erythroids, monocytes, and granulocytes. The Krumsiek et al. model (44) is a Boolean network model of common myeloid progenitors transitioning into megakaryocytes, erythrocytes, granulocytes, and monocytes. The two models share 6 genes. Importantly, both models include key AML-related genes absent in the other model; the Bonzanni et al. model includes *RUNX1* while the Krumsiek et al. model includes *CEBPA*, both of which are involved in the pathogenesis of AML (45,46). Merging these models results in a more detailed explanation of the HSPCs differentiation process and thus possibly a more comprehensive representation for AML patients with different genetic alterations.

In Fig 2(b), the Palma et al. model (14) integrates patient-specific genomic data into a Boolean network to predict clinical outcomes in AML patients, focusing on key regulatory pathways and mutations frequently observed in AML. The Ikonomi et al. model (47), on the other hand, uses a Boolean network to model HSC maintenance and the regulation of the *TP53* pathway in response to niche interactions. These models overlap in their representation of key regulatory pathways involved in hematopoiesis and AML. By merging these models, we can gain a deeper understanding of how genetic mutations in AML impact both the differentiation of blood cells and their interactions with signals from the stem cell environment, providing insights into the disease’s progression and potential therapeutic targets.

### Evaluation of the merged models

Next, we demonstrate the potential of model integration to provide a more comprehensive understanding of complex biological processes, particularly in diseases like AML where multiple regulatory pathways and genetic factors are involved. We merged each of the selected pairs using 3 methods: the OR Combination, the AND Combination, and the Inhibitor Wins Combination (see Methods section for details). To assess the effectiveness of our proposed workflow, we evaluated the merged models against the original studies and tested their ability to reproduce key findings and generate new insights about AML.

### Evaluation of the merged models using steady states analysis

**Fig 3:**
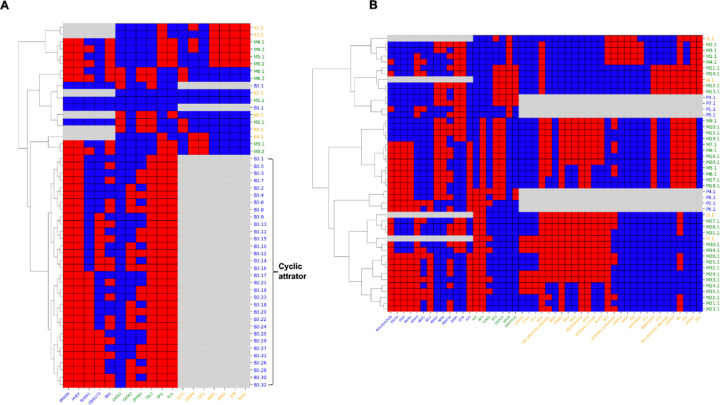
Steady states pattern of the merged models. (A) Steady states pattern of the Bonzanni - Krumsiek merged model pair and the individual models. The color in the heatmap indicates that a gene is ON (Blue), OFF (Red), or that the gene is not included in the model (Grey). For the x-axis labels, blue and orange indicate genes of the individual models (blue: Bonzanni et al. model, orange: Krumsiek et al. model), and green indicates genes shared by both models. For the y-axis labels, blue and orange indicate steady states of the individual models (blue: Bonzanni et al. model, orange: Krumsiek et al. model), and green indicates steady states of the merged model. (B) Steady states pattern of the Palma - Ikonomi merged model pair and the individual models. Here blue labels indicate genes or states from the Palma et al. model, and orange labels indicate genes or states from the Ikonomi et al. model.

We performed the steady states analysis using the asynchronous updating method for both model pairs (Fig 3a, the Bonzanni2013-Krumsiek2011 pair; 3b, the Palma2021-Ikonomi2020 pair) to identify all possible attractors including the cyclic attractors. To evaluate the consistency and robustness of the merged model, the attractors patterns of both the individual models and merged models are clustered based on the hamming distance between them. For the merged models, results of the Inhibitor Wins Combination models are shown in Fig 3, and results of other combination methods can be found in Fig S1.

A common goal of the Bonzanni et al. model and the Krumsiek et al. is to describe cellular behavior of HSCs, and they have both compared the steady states of their models with experimental data. It has been reported that each of the individual models can generate states that are representative for different cell types originated from HSCs (39,44). Our results demonstrated the reproducibility of the original papers with the same stable states. We also found states B2.1 and K5.1 shown in Fig 3 are representative for the erythroid cells as characterized in the original papers. One interesting result is that the merged model also captures such behavior and combines them in a single state M2.1, where the expression of *Hhex*, *Runx1* and *Erg* are inactive and *Gata1*, *Tal1* and *Klf1* are active. This has been demonstrated by microarray experiments of erythroid cells (48), where the active genes have a higher expression and the inactive ones have a lower expression. (See Fig S2 for the comparison.) Additionally, an interconnected cyclic attractor that consists of 32 states is identified and clustered in the original Bonzanni et al. model, shown on the bottom of Fig 3a, including a state that represents the expected pattern of HSPCs (B3.32). This cyclic attractor suggests that HSPCs consist of a heterogeneous cell population. The steady states derived from our merged model (M3.1 and M3.2) are clustered together with the cyclic state, which feature multiple genes active and *GATA1* consistently repressed. In contrast to the Bonzanni et al. model, cyclic attractor M3.1 and M3.2 of the merged model do not show a variability in *ERG* and *GATA1*, suggesting their unique roles in HSC maintenance (49,50).

Similarly, for the Palma2021-Ikonomi2020 model pair, the merged model replicates the steady states of the original models. As shown in Fig 3b, each steady state from the individual models corresponds to several steady states in the merged model. Take an example, attractors I1.1, M1.1-M4.1 (the first 5 rows of Fig 3b) represents the long-term HSCs where genes related to metabolism or cell growth are repressed (*MTOR*, *ROS*, *MYC*, *SOX4*, *MAPK1*, etc.), and cell cycle inhibitors are activated (*CDKN1C*, *CDKN1A*, *CDKN1B*, *GFI1*), as well as *ROS* and *MTOR* signaling, which has been observed in quiescent HSCs. Furthermore, the merged model also provides a more comprehensive view of gene regulation. For example, both original models only capture part of the regulations of the *TP53* tumor suppressor gene: the Palma et al. model states that *ARF* (*CDKN2A*) activates *p53* (*TP53*); and the Ikonomi et al. model describes the negative feedback of *MDM2*. Integrating the models provides a more comprehensive view of *p53* regulation, where *ARF* activates *p53* through the inhibition of *MDM2* (51).

In summary, the steady states analysis demonstrates that the merged models effectively capture the behaviors of the individual models. This validation supports the integration methodology, ensuring that the merged models maintain the biological relevance and functionality of the original gene regulatory networks. Importantly, the merging process increases the coverage of genes and interactions, generating a more comprehensive model for further analysis and exploration.

### Evaluation of the merged models with gene expression data

**Fig 4:**
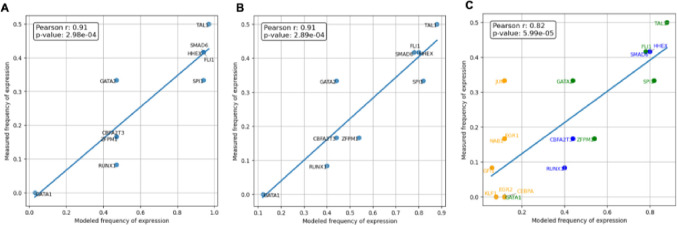
Expanding gene behavior modeling through merged models. (A) Correlation of averaged gene expression from the 12 single-cell profiles of HSPCs from a previous study (52) to the average gene expression from the modeled steady states for each of the 10 genes in the Bonzanni et al. model. The blue line shows the linear regression model fit. (B) Correlation of the gene expression data with the average gene expression from the steady states of the 10 genes using the merged model. (C) Correlation of the gene expression data with the average gene expression from the steady states of 18 genes using the merged models. 8 additional genes not covered in the Bonzanni et al. model are colored in orange, together with genes covered in the Bonzanni et al. model only (blue) and shared by both models (green). Scatterplot of the ‘AND’ model are shown, for other merged models results, see Fig S3.

A key finding from the Bonzanni et al. study is the prediction power of the network model for gene expression profile. The modeled frequency of expression for each gene is calculated as the proportion of steady states in which the gene is ‘on’ (Boolean state of 1) relative to the total number of steady states in the model. Similarly, the measured frequency of expression can be derived from the proportion of cells expressing the gene in single-cell microarray experiments of HSPCs (52). Results of the Bonzanni et al. model show a near-linear correlation of the expression frequency in the experiments to the modeled frequency of expression for each of the genes (39). For the evaluation of the model reproducibility, our analysis achieved comparable results as the original study, with a Pearson correlation coefficient of around 0.9 (Fig 4a). We perform a similar analysis using the merged models for the genes appearing in the Bonzanni model, and our results show the merged model can achieve a similar correlation coefficient (Fig 4b), suggesting a comparable prediction power of the network model for gene expression profile.

We then asked whether the merged model can be predictive for the gene expression profile to a wider range of genes. We apply this analysis to an extended set of genes in the merged model by including additional genes covered in the Krumsiek et al. model (Fig 4c). To make the results comparable, the same gene expression data used in Bonzanni et al. was used for this analysis, including the expression of 8 additional genes covered in the merged model. The results show that the expression from a wider range of genes correlates well with the modeled steady states of the merged models, with a Pearson correlation coefficient of 0.82. We observed a declined correlation between the measured and modeled frequency of gene expression derived from the Krumsiek et al. model. This might be due to the inconsistency of the measured data which derived from the HSPCs, as the model is more specific to simulate common myeloid progenitors which are in a later stage of HPSC differentiation. For example, *KLF1* is essential for erythroid lineage commitment and is not expressed in HSCs (53), and *GFI1* is involved in myeloid differentiation(54); *CEBPA* is crucial for myeloid progenitor differentiation, and has been shown to have a relatively low expression level in HSCs (55).

### Evaluation of the merged models with clinical outcomes

The evaluation of the merged Palma-Ikonomi models focuses on their ability to predict clinical outcomes for patients with specific mutations. Palma et al. have connected their network to three cancer hallmark phenotypes: apoptosis, differentiation and proliferation. In addition, an integrated network score was defined as subtracting the value of apoptosis and differentiation from the value of the proliferation to reflect the malignancy of the cancer. The phenotype scores were then used as a proxy of the model to patients’ clinical outcomes (14). The two clinical outcomes of interest are (1) the impact of mutations on overall survival, as measured by the Cox hazard ratio; and (2) the progression of disease, as measured by the percentage of blast cells.

To evaluate the reproducibility of the model, we first analyzed the correlation between the simulation outputs of the Palma et al. model and (1) the mutation-specific hazard ratios from a clinical study (AMLSG (21)) and (2) the blast percentages of AML patients from the TCGA-LAML dataset (15). Then the merged models were used to run a similar analysis for comparison. The results are summarized in Table 2, for details, see Fig S4 and S5. The findings indicate that the merged models show strong correlations with hazard ratio for death, comparable to the original Palma et al. model. This suggests that the integration methods effectively capture the clinical implications of the genetic mutations, and implies that a more stringent approach would be more promising for clinical outcomes prediction.

**Fig 5:**
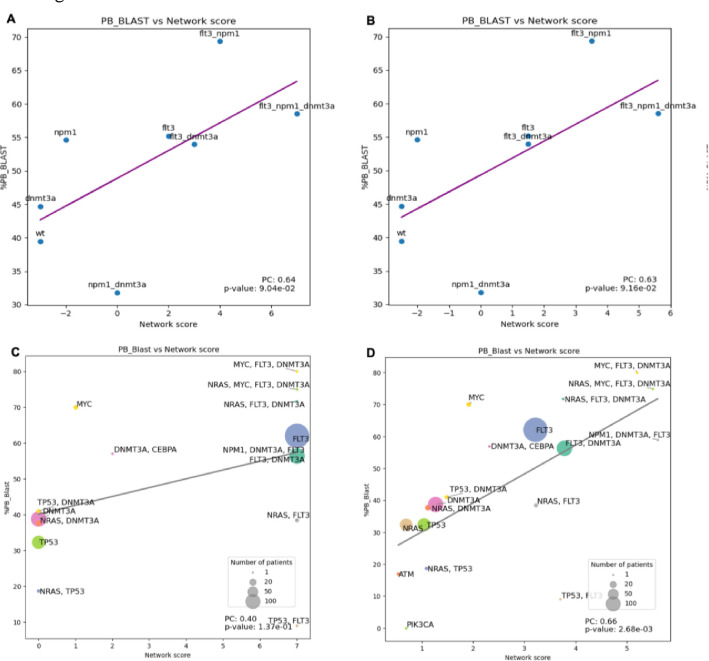
Expanding clinical outcome modeling through merged models using the Beat AML dataset. The Beat AML, a large AML dataset, was used to evaluate the models’ performance on a more diverse patient population. (A) Correlation between the average blast percentage in peripheral blood and network scores derived from the Palma et al. model for different mutation status on *FLT3*, *NPM1* and *DNMT3A*. (B) Correlation between blast percentage and network scores derived from the merged model for different mutation status on *FLT3*, *NPM1* and *DNMT3A*. (C) Correlation between the average blast percentage and network scores derived from the Palma et al. model for different mutation status on all available genes in the Palma et al. model. Size of node indicates number of patients for each mutation profile. (D) Correlation between the average blast percentage and network scores derived from the merged model for different mutation status on all available genes in the merged model. Only scatterplot of the ‘AND’ model are shown, for other merged models results, see Fig S6.

Further, to evaluate the robustness of the models, we applied the model to a different, larger AML dataset, Beat AML, which includes genomic and clinical data for 805 AML patients with 942 specimens (18). Using the Beat AML dataset, the correlation between the Palma et al. model’s network score and blast percentage in peripheral blood (PB_blast) is 0.64 (Fig 5a) and for blast percentage in bone marrow (BM_blast), the correlation reaches 0.71. For the merged models, results are comparable, with the correlation for PB_blast reaching 0.63 (Fig 5b) and the correlation for BM_blast reaching 0.72 (Fig S5). Our results demonstrate that the models show robustness across two datasets, for both the original model and the merged model. Detailed results for both blast percentage in peripheral blood (PB_blast) and bone marrow (BM_blast) are shown in Fig S6.

Palma et al. identified patients’ mutation profiles as the mutation status of only three critical AML genes: *FLT3*, *NPM1* and *DNMT3A*. One limitation of the approach is that patients with mutations in other genes, which may play an equally important role in AML prognosis, are not well-represented in the analysis. For example, 9.4% of the patients in the Beat AML dataset (76/805) carry at least one mutation in *TP53* gene, but such mutations are ignored in the Palma et al. analysis. A goal of model merging is to capture a more comprehensive AML gene regulation landscape, therefore, we next identified patients’ mutation profiles using all available genes covered by the model and tested the models’ prediction on their blast percentage for both models.

Fig 5c and 5d show the relationship between network scores and the average percentage of blast cells in bone marrow for patients with each mutation profile. Performance of the Palma et al. model declined when expanding the coverage (Pearson correlation: 0.40, p = 0.14). The merged model improved the prediction power, with the “AND” merging model showing a correlation of 0.66 and a p-value of 0.0027. This improvement can be attributed to the richer information captured in the extended model, which accounts for patient populations with less common mutations, as indicated by the smaller data points. For example, clinical responses of the *TP53*-mutated patients can be modeled more accurately using the merged model, as shown by the light green circles in Fig 5c and 5d. Since the Ikonomi et al. model describes the regulation of the *TP53* pathway in more detail, it compliments the Palma et al. model, which only includes high-level interactions of the AML hallmark genes.

Together, these results demonstrate that merging models not only preserves but enhances the predictive accuracy of GRNs for AML patients. This underscores the potential of integrated models to guide treatment strategies by accurately simulating the complex interactions and outcomes based on patient-specific genetic profiles.

## Discussion

One significant reason for merging gene regulatory network models is to increase the coverage of patients with different mutations, thereby enhancing the model’s applicability. By integrating models, we can cover a broader range of genetic variants found in AML patients. By investigating the genetic profiles of AML patients from AMLSG (21) and cBioPortal (56) (Fig S7), we found that merging the Palma-Ikonomi models increases the coverage of 285 patients with mutations in *TP53*, a significant tumor suppressor gene which is not covered in the Palma et al. model analysis. Similarly, the merged Bonzanni-Krumsiek models show an increase of 255 patients for *CEBPA* mutation. The implications of covering these mutations that might not be adequately represented in individual models are significant. By including more mutations in our models, we can enhance the ability to develop personalized therapeutic strategies. This expanded coverage ensures that our models are more comprehensive and better reflect the genetic diversity observed in AML patients.

Efforts have been made to model GRNs from a broader view. Tools like RegNetwork (57) and NDEx (Network Data Exchange) (58,59) combine GRNs by leveraging shared regulatory components from multiple sources. These super-networks provide insights of gene regulation and have been shown useful to investigate context-specific mechanisms. However, these tools are primarily designed for storing, sharing, and visualizing biological networks, but do not typically offer computational tools needed for quantitative analysis of GRNs. As another example, researchers who aim to do “whole cell modeling” (WCM) also aim to integrate biosimulation models. WCM aims to simulate the entirety of cellular processes, including gene regulation, metabolism, and signal transduction, by integrating various types of biological networks (60). Despite the ambitious vision, currently WCMs are only available for a few organisms, and most of them are bacteria like *M. genitalium* (61) and *E. coli* (62) that have small genomes and are well-studied. The collection and integration of heterogeneous data remains to be the major challenge, and this need continuously grows with the complexity of the cell (63,64). Also, these efforts are mostly focused on ODE and rule-based models, and there is currently no integrator specifically designed for logical models. This gap underscores the need for a framework that can effectively merge logical models to enhance their predictive power and applicability in understanding complex biological systems.

Logical models have been recognized as the simplest form of GRN modeling and thus the easiest to scale-up. While this is a simplification of the continuous genetic behavior, the sigmoidal shape of regulatory interactions supports the assumption of discrete states (33). The impact of a regulator on the activation of its target is marginal before reaching a certain threshold concentration, and rapidly increases to a maximal rate afterwards. The sensitive behavior was also observed in signal transduction where the phosphorylation and dephosphorylation modification that regulates the activity of a protein are in sigmoidal shape given the Michaelis-Menten kinetics (65). These observations justify the assumption that the level of gene or protein activity is discrete, which helps simplify the problem a lot while preserving its high accordance with reality (6). Therefore, we here use logical models to demonstrate the integration of multiple GRNs as a starting point, and aim at providing a workflow to model the higher-level behavior of biological systems.

Here, we have proposed and tested three deterministic methods for model merging, namely ‘AND’, ‘OR’ and ‘Inhibitor Wins’ (see Methods section). As an alternative, one could also use a probabilistic approach, where probabilities are assigned to each logical rule from the individual models (32,66). In biology, gene expression and TF binding have a stochastic nature, which is reflected in the random and context-dependent interactions among biological entities (67,68). Therefore, a probabilistic approach might better model the uncertainty and variability in regulatory interactions. In general, researchers will have to carefully consider how to merge rules from different models, depending on their research objectives and biological contexts.

This model merging workflow can be used to construct more comprehensive mechanistic models for diseases, which is one key aspect for biomedical digital twins (69). A digital twin is a set of virtual information constructs that mimics the structure, context, and behavior of the physical twin, and is dynamically updated with data from its physical twin (70). The model merging workflow allows researchers to systematically construct a comprehensive virtual representation of a disease (the physical twin) by integrating previously developed models. We argue that the merged models provide candidate models for biomedical digital twins, which in combination with pertinent patient data can be used to generate patient-specific models for predicting response to personalized treatments. In the case of AML, model personalization is possible by integrating mutation states of patients and drug response data available in datasets such as Beat AML. These personalized models could be used to test “in silico” for individual responses to drug interventions. Moving beyond AML, logical model merging could be applied in many other contexts wherever researchers have developed logical models of biological processes.

To our knowledge, this is the first study on logical model integration, and it still requires further improvements. The major obstacle is the lack of comprehensive, high-quality data for certain genes and interactions, which can limit the accuracy evaluation of the merged models. Although repositories for curated data and logical models exist, they are primarily for internal validation and are not easily mapped to other external models (71). Moreover, extensive annotation is required to determine the biological relevance of the models, including the knowledge source and primary purpose of the model. This underscores the importance of developing systematic documentation and annotation standards as a community effort. The reproducibility of models is another critical aspect. In our study, we evaluated the reproducibility of each individual model before merging, and then compared the performance of the merged models with the original models across datasets. Using the Palma et al. model, we have analyzed its correlation with hazard ratios and the blast percentage of AML patients as in the original publication (Fig S3, S4). Despite a minor discrepancy in the Palma et al. model, most of the selected models are reproducible. This highlights the importance of ensuring the reproducibility of the models in order to provide reliable predictions and support their applicability in broader research and clinical contexts.

Moving beyond logical formalism, future work could expand this workflow to incorporate more complex model types, which would enable us to model broader biological contexts. One possible direction is the integration with ODEs, which provide a quantitative description of the biochemical processes involved. There are various studies on the transformation (‘odefy’) from discrete logical models to continuous ODE models where the dynamic descriptions can be derived automatically from logical rules (72,73). Based upon that, a hybrid approach can be used where discrete states from logical models trigger continuous dynamics modeled by ODEs. The constant sign property has been proposed to determine whether an ODE model is consistent with a signed directed graph, which could be used to facilitate the integration of discrete and continuous dynamics (74).

In summary, this study presents a workflow for integrating logical models of GRNs, using AML as a case study. We demonstrated the effectiveness of our approach by merging two pairs of AML-focused models, capturing the complex interactions and regulatory mechanisms involved in gene expression and clinical outcomes. Our merging methods, including ‘OR’, ‘AND’, and ‘Inhibitor Wins’, align well with biological phenomena and provide robust predictions, as evidenced by the strong correlations with experimental and clinical data. Our work also underscores the importance of standardization, reproducibility, and systematic documentation in logical models, and calls for comprehensive, high-quality data and extensive annotation. By addressing these challenges, the workflow has the potential to advance our understanding of complex biological systems and support the development of more effective and personalized therapies for diseases like AML.

## Methods

### Logical GRNs models

Mathematical models have been developed to capture the behavior of GRNs, among them, logical models are one of the simplest and most frequently used by biologists. This is partly due to limited information on kinetic parameters besides network structure, but primarily because logical models are intuitive and versatile (3,4). Despite their simplicity in formulation, logical models are able to generate and recapitulate complex behaviors often observed in cellular biology. Logical models use directed hypergraphs to describe not only pairwise relationships, as interaction graphs do, but also interactions between more than two components, which is common in biochemical reactions and signaling transductions (6). Since Kauffman’s study in 1969 (7), logical models have been used to describe a wide range of activities in biological systems, from gene activities and protein expression to cellular behavior and system-level phenotypes, ultimately contributing to clinical and translational medicine (For reviews, see (2,4,8,9)).

Logical GRN models are represented as directed graph G=(V,E), where V is the set of nodes representing genes or proteins, and E is the set of edges representing regulatory interactions. Each node $v_{i}\in V$ can take on a state $s_{i}$ from a finite set S, where $s_{i}\in S$. For Boolean models, S={0,1}, where 0 represents an inactive (OFF) state and 1 represents an active (ON) state; and for multi-value logical models, S={0,1,…,k}, where level of activation can be specified. Time is viewed as proceeding discretely in general logical models. States of the nodes can be updated synchronously, when all values are calculated after a transition, or asynchronously, with one at a time. At each step, the state of each node $v_{i}$ is determined by a logical rule $f_{i}$, which is a function of the states of its regulators. Formally, for each node $v_{i}$:

$$s_{i}(t+1)=f_{i}\left(s_{1}(t),s_{2}(t),\ldots ,s_{n}(t)\right)$$

where $s_{i}$ denotes the state of node $v_{i}$ at time t, $s_{l}$ through $s_{n}$ are the regulators of $s_{i}$, and $f_{i}$ is a logical function defined by logical operators.

These logical functions are the core of the network and define its behavior. Using a combination of logical operations, namely AND, OR and NOT, we can then describe the updating schema for a gene or any biological molecule (33). For standardization and convenience, we use the extended Backus–Naur form to represent the network (75). We restrict the logical operators to logical product (AND, represented by the symbol ‘&’), logical sum (OR, represented by the symbol ‘|’), and logical negation (NOT, represented by the symbol ‘!’). To illustrate this notation, consider the regulation of MYC, a critical oncogene. MAPK signaling positively modulates MYC expression and activity (76), while FBXW7 targets MYC for proteasomal degradation with GSK3B required for its recognition through phosphorylation (77,78). Therefore, the logical function of the activation of $MYCf_{MYC}$ can be described as:

$$f_{MYC}=MAPK1\;\&\;!(FBXW7\;\&\;GSK3B)$$

To translate this formula into English: MYC is turned on if both MAPK1 is “on” and at least one of FBXW7 and GSK3B is “off”. This symbolic representation is particularly useful in understanding the ordered activation and inhibition of GRNs, where inputs like regulators and signals operate jointly in a cascade (3).

### Workflow for model integration

#### Finding models

The initial phase of integrating logical models involves identifying candidate models for integration. This typically starts with utilizing existing data like publications and repositories relevant to the biological system under investigation. Researchers often look into large repositories of biological logical models, such as the Cell Collective(79), the GINsim repository(80) and BioDiVinE(81), to find logical models of interest.

The impetus for adopting logical models frequently arises from the need to obtain a mechanistic understanding of biological systems that, due to their complexity, insufficient data, or both, render reaction-based modeling impractical. Examples of such systems include complex pathways, cellular processes, or disease mechanisms. Pathway databases like KEGG(82), Reactome(83), SIGNOR(31), etc. are also useful resources although they only provide interaction graphs and need to be translated into logical relationships afterwards. To generate logical models from pathway data, one must first translate the interaction graphs into logical rules that define gene and protein interactions. An effective approach to this translation is to use the ‘Inhibitor Wins’ combination method, where inhibitory interactions dominate over activatory ones (See the following section). However, it is crucial to carefully review the literature for supporting evidence to ensure the logical combinations accurately represent biological mechanisms.

After gathering logical models and building a comprehensive library of potential models, each model should be carefully reviewed to assess its relevance based on the topic, components involved, and the knowledge source. The decision should be primarily based on the purposes of the models and their biological relevance. Another important consideration is the amount of overlap, and we aim for the models to complement each other by expanding the coverage of nodes while ensuring that they share key regulators. Models that share components and are biologically relevant are chosen for further analysis. This selection is critical to ensure that the integrated model accurately and comprehensively represents the underlying biological processes and can provide meaningful insights. Ensuring sufficient overlap while also expanding the node coverage enhances the model’s robustness and its ability to simulate complex biological interactions effectively.

#### Standardizing & annotating models

Reproducibility, a long-standing concern of the scientific community, can be significantly improved through the use of standards, annotations and repositories (84). The accurate and appropriate description of both the model itself and its components is also essential for model composition. As proposed by the curation and annotation of logical models (CALM) initiative (85), we use the Systems Biology Markup Language Qualitative Models (SBML-qual) format (24,25) to represent the selected models.

Standardized gene naming is crucial for promoting research, and the Human Genome Organization (HUGO) was established to guide this process (86). The community often refers to the HUGO Gene Nomenclature Committee (HGNC) guidelines for naming genes, which include not only protein-coding genes but also RNA genes and pseudogenes. Additionally, orthologs across vertebrate species are encouraged to use the same gene symbol, such as for mouse and rat. To ensure accurate gene identifiers and facilitate the later composition process, we mapped each gene in the models to the HGNC gene ID (26) and annotated them with the HGNC approved symbol. For proteins, it is recommended to use the same abbreviation as genes, and the standard nomenclature should be referred to the International Protein Nomenclature Guidelines (87). Special attention should be given to fusion proteins and complexes, for which standard nomenclature may not exist. Metadata including original sources of the models should also be included in the annotation.

We also propose linking each component in the models to online resources like NCBI Gene and ChEMBL, and annotating the supporting evidence of the edges. This involves documenting the type of interaction (e.g., activation, inhibition), the experimental method used to determine the interaction, and the source of the data (e.g., specific publications or databases). This level of detailed annotation ensures that the models are transparent, reproducible, and easily integrated with other datasets, thereby enhancing their utility and reliability in further research and applications.

#### Reproducing selected models

Reproducibility is believed to be one of the central pillars of science. However, as the models become more complex, the task becomes more difficult (41,42). There has been a longstanding crisis of reproducibility in computational biology, as a study investigating 455 published kinetic models from the BioModels, half of the model results could not be reproduced (88). The degree of reproducibility is often described based on how much of the original material is used to recreate the model study (84). Here, we focus on whether we could obtain the behavior of the model as described by its authors to ensure the credibility of the model.

Evaluating the selected models begins with performing simulation experiments as described in the original sources. Each model is simulated independently using the same conditions and parameters specified in its original publication. The results are then compared and validated against the claimed performance in the original studies. This validation step is essential to ensure that the models are correctly implemented. Additionally, the interoperability of the standardized models can be verified by cross-platform testing, ensuring that the models produce consistent results across different computational tools and environments. To facilitate the task, the CoLoMoTo Interactive Notebook has been developed by the Consortium for Logical Models and Tools (CoLoMoTo) to provide an easy-to-use environment to perform analysis of biological networks, where logical modeling tools are integrated in a unified interface (89).

#### Composing models

The composition of multiple logical models involves identifying the overlapping and non-overlapping components of the models. Let $M_{1},M_{2},\ldots ,M_{n}$ be the models to be integrated. The sets of nodes and edges for each model are denoted $\left(V_{1},E_{1}\right)\left(V_{2},E_{2}\right),\ldots ,\left(V_{n},E_{n}\right)$.

First, we identify the overlap O and non-overlap $N_{i}$ components:

$$O=\overset{n}{\underset{i=1}{⋂}}V_{i}$$

$$N_{i}=V_{i}\backslash {⋃}_{j\neq i}^{⬚}V_{j}$$

For the overlapping components, the logical functions $f_{i}$ for each node $v_{i}\in O$ need to be updated. The merged logical function $f_{i}^{\mathrm{merged}}$ is defined using logical combination methods. We employ three methods, each with a unique rationale for merging the logical rules that govern gene or protein interactions:

##### 1. OR Combination: $f_{i}^{OR}={⋁}_{j=1}^{n}f_{i}^{M_{j}}$

This method combines the logical rules from the individual models using the logical OR operator. If either of the rules from the models predicts the activation of a node, the combined rule will also predict activation. This approach ensures that the integrated model captures all possible activation scenarios, providing a more inclusive representation of the regulatory network.

##### 2. AND Combination: $f_{i}^{AND}=\Lambda_{j=1}^{n}f_{i}^{M_{j}}$

In contrast, the AND combination method uses the logical AND operator to merge the rules. Here, a node will only be activated in the integrated model if both original models predict its activation. This method is more stringent, ensuring that only consistent activation predictions are retained, which can reduce false positives and emphasize strong, corroborated regulatory interactions.

##### 3. Inhibitor Wins Combination: $f_{i}^{IW}=\left\{\begin{array}{ll} 0 & \;\text{if}\;\exists j\;\text{such}\;\text{that}\;f_{i}^{M_{j}}\;\text{contains}\;\text{any}\;\text{active}\;\text{inhibitor}\;\\ max\left(f_{i}^{M_{1}},f_{i}^{M_{2}},\ldots ,f_{i}^{M_{n}}\right) & \;\text{otherwise}\; \end{array}\right.$

This method prioritizes inhibitory interactions. If any edge in the original models represents an inhibitory relationship, this inhibition will dominate in the merged model, leading to the node being turned off or having a negative impact on its status. This approach reflects the biological reality where inhibitory signals often have a strong regulatory effect, such as in the suppression of oncogenes or other critical pathways (14,90).

By employing these combination methods, we can then select the one that best fits the goal of composition based on biological considerations and the specific requirements of the study (inclusivity, stringency, or regulatory dominance). For the non-overlapping components, the nodes and their associated logical functions are directly added to the merged model. The final merged model $M_{\mathrm{Merged}}$ consists of:

$$V_{\mathrm{merged}\;}=O\cup \cup_{i=1}^{n}N_{i}$$

$$E_{\mathrm{merged}}=\overset{n}{\underset{i=1}{⋃}}E_{i}$$

There is strong evidence supporting our proposed logical combination methods for merging GRN models. Given that gene regulation can be viewed as the interplay of transcription factors (TFs) and TF-binding sites of mRNA to govern expression levels of mRNA and their resulted proteins (91), it is intrinsically related to enhancer function and logic (92,93). Combining rules using the ‘OR’ operator aligns with the concept of enhancer integration where multiple TFs can independently activate a gene, enhancing robustness and flexibility. The ‘AND’ method, requiring all conditions to be met for activation, mirrors cooperative binding of TFs, ensuring specificity and fine-tuned control of gene expression. The ‘Inhibitor Wins’ method, where inhibitory interactions dominate, reflects the biological reality where repressive signals prevent inappropriate gene expression. This is supported by studies on transcriptional repression, where inhibitors can override activatory signals (94).

While the OR and AND rules are straightforward for Boolean models, they become more complex for multi-value logical models due to differing scales. To address this, harmonizing the scales of the models before composition is essential. This involves converting all models to a common scale that allows for consistent interpretation of the logical functions. One approach is to normalize the values in each model to a standardized range, ensuring compatibility during integration.

Once a merged model has been built, we encourage researchers to make this new model readily available in a public repository, such as the Cell Collective (79) or the GINsim repository (80). Ideally, researchers should document their approach to merging so that the process is reproducible. Sharing and discussion of these merged models with the scientific community could then foster new collaboration, and enhance the quality and applicability of these merged models.

#### Evaluating the merged model

The aim of evaluation is to ensure that the integration maintains the biological relevance and functionality of the original models. The first step is to replicate the conditions and parameters of the original studies using the merged model. By comparing these simulation results with the original outcomes, we verify if the merged model retains the predictive accuracy of the individual models. Next, we test the merged model on new tasks not included in the original studies to assess its robustness and ability to generalize to different biological scenarios, such as predicting gene expression under new conditions or the impact of novel mutations.

To identify the optimal combination method, we evaluate the performance of the merged model using different integration strategies. By comparing these methods, we determine which approach best captures the biological processes and provides the most accurate predictions. The method that offers the highest accuracy and predictive power while aligning with the biological context of the study is then selected to finalize the merged model.

#### Application to logical model composition for AML

We use AML-focused logical models to exemplify the model composition workflow, and evaluate performance of the merged models against original studies.

##### Finding AML logical models.

We conducted a comprehensive literature search on PubMed to identify previously developed logical models related to gene regulation in AML. Specific search terms such as “AML gene regulatory network,” “AML Boolean network model,” and “AML qualitative network model” were used (see table S1 for the complete list of keywords). Additional relevant papers were later identified through manual searches on Google Scholar and bioRxiv, addressing gaps not covered by the initial search terms. The complete list of keywords used is provided in Table S1. The initial search highlighted the need for manual review to identify truly relevant models, as studies sometimes use less specific terminology. For example, papers using terms like “signaling networks” or “signaling pathway” were not captured by the keyword search alone, while adding those keywords result in more irrelevant papers on function of one specific gene or protein. Rigorous selection criteria were applied to ensure the relevance of the identified models. Models had to be AML-related and employ Boolean or qualitative logic. Preference was given to models validated with patient data or experimental evidence. Finally, the model pairs for merging should share an adequate level of overlapping genes.

##### Standardizing & annotating the AML logical models.

Next, we standardized and annotated these selected models in SBML-qual format. Each model was annotated using the HGNC resource to ensure accurate and consistent gene and protein identifiers.

##### Reproducing model results.

The reproducibility of these models is validated against their original publications using different computational tools including R, Python, and GINsim. Detailed methods and parameters used can be found in the original studies. Subsequent simulations and analyses were conducted in the CoLoMoTo Interactive Notebook using docker image colomoto/colomoto-docker:2024–03-01.

##### Composing AML models.

Three approaches are tested for combining rules: the OR Combination, AND Combination, and Inhibitor Wins Combination as defined previously.

##### Evaluating the merged models.

Each approach is validated for performance against original studies. Steady states analysis using the asynchronous updating methods is performed for each of the model pairs. The expression patterns from the steady states of merged models are hierarchical clustered together with the steady states of individual models for comparison, and the nearest neighbor approach using Hamming distance was implemented for clustering.

Further, the stable states from the Bonzanni - Krumsiek model pair are compared with gene expression data of blood cells generated by Chambers et al. Data was obtained as GCRMA normalized microarray data. We calculated the average expression level of each cell type and converted them into dichotomous variables to compare with our Boolean states.

To demonstrate benefits of the model composition, for the first model pair, we correlate the averaged gene expression activity from single-cell profiles of hematopoietic stem cells (HSCs) (52), as used in one of the original studies, with the average gene activity from modeled steady states in both individual and merged models. For the second model pair, we identified patients’ mutation profiles as the mutation status of three critical AML genes: *FLT3*, *NPM1* and *DNMT3A*. Phenotype scores were calculated for each profile as described by Palma et al. (14) and correlates with clinical outcomes: hazard ratio of death from the AMLSG dataset (21) and blast percentages from the TCGA-LAML dataset (15).

To cover the patients with other mutations, which could not be modeled using the previous approach, we tested the models’ performance using all mutations available in the Beat AML dataset. E.g., the Palma et al. model covers 18 genes, if a patient has any of them mutated, the combination of those genes would be used as his/her mutation profile. A similar approach was done for the merged model, except more genes are included. We identified the mutation profile of each patient using the mutation calls data and applied filters on the SIFT score (95) and PolyPhen score (96) to find only the deleterious variants. In addition, *FLT3-ITD* mutations derived from clinical genotyping results are added. For each profile, the phenotype scores are calculated as previously and compared with the average blast percentages.

### Implementation

Models used in the study are provided in Supporting information as both text files and SBML-qual files.

All codes used for running experiments, model merging, and evaluation are available on a GitHub repository at https://github.com/IlyaLab/LogicModelMerger/.

### Materials

The following public data are used in the evaluation of the merged models:

1. Gene expression data of HSCs and their differentiated progeny using microarray analysis by Chambers et al. (48). Data was taken from Table S2. Complete GCRMA Normalized Hematopoietic Microarray Data Set. in the supporting information of the publication.
2. The averaged gene expression activity from single-cell profiles of HSCs provided by Ramos et al. (52). Data was taken from Table S6: MAS Calls in Each of the Samples Amplified with GSC RT-PCR Followed by Oligonucleotide Microarray Analysis in the supporting information of the publication.
3. Hazard ratio of death given by a clinical study of 1540 AML patients (AMLSG), where survival analysis was performed using Cox proportional-hazards methods (21). Mutation-specific hazards for *FLT3*, *NPM1*, *DNMT3A* and their combinations are given in Table 2 in the publication.
4. Blast percentages and genetic mutations of 200 AML patients from the TCGA-LAML study, where whole-genome sequencing (50 cases) or whole-exome sequencing (150 cases) are performed and clinical outcomes are collected (15). Mutation and clinical data were obtained from the NIH GDC website (https://gdc.cancer.gov/about-data/publications/laml_2012).
5. Blast percentages and genetic mutations of 805 AML patients from the Beat AML study, where a custom capture library (using whole-exome sequencing identified mutations) were used to generate the mutations of the patients. Mutation and clinical data were obtained from the BeatAML2 website (https://biodev.github.io/BeatAML2/).

## Supplementary Material

Supplement 1

## Figures and Tables

**Table 1:** Summary of the identified AML-related logical models for merging.

Models	First Author	DOI	Model Type	Topic	Purpose	Knowledge Source	Verification	Model Availability

Pair #1	Palma A	10.3390/jpm11020117	Boolean network	AML	Clinical outcome prediction	Literature review	*In silico*	Full description in text.
	Krumsiek J	10.1371/journal.pone.0022649	Boolean network	Myeloid differentiation	Understand cellular behavior	Literature review	*In silico*	Full description in text.
Pair #2	Ikonomi N	10.3389/fphys.2020.00848	Boolean network	HSC homeostatic maintenance	Understand cellular behavior	Literature review	*In silico*	Full description in text.
	Bonzanni N	10.1093/bioinformatics/btt243	Boolean network	Early blood stem cell development	Understand cellular behavior	Literature review	*In silico & in vitro*	Full description in text.

**Table 2:** Summary of the correlation with clinical outcomes using the AMLSG for hazard ratio and TCGA-LAML data for blast percentage.

		Palma et al. model	Merged model with ‘OR’ rules	Merged model with ‘Inhibitors win’ rules	Merged models with ‘AND’ rules

**Hazard ratio for death**	Correlation	0.73	0.73	0.66	0.73
	P-value	0.041	0.041	0.077	0.040
**Blast percentage in peripheral blood**	Correlation	0.71	0.68	0.69	0.67
	P-value	0.050	0.063	0.057	0.067
