# Supplementary material for: Merging logical models: An application in Acute Myeloid Leukemia modeling
Source: bioRxiv. 2024 Sep 19:2024.09.13.612961. Preprint. [Version 1] doi: 10.1101/2024.09.13.612961 (PMC11429764; doi:10.1101/2024.09.13.612961)
Supplement: Supplementary file 1 [file media-1.pdf]

Merging logical models: An application in Acute Myeloid Leukemia modeling

Supporting information

Table S1: Keywords used in literature retrieval for AML-related logic models.

Keywords in the same category are combined using OR, and the two categories are combined using AND.

Category	Keywords
Disease Related	Acute Myeloid Leukemia<br>AML<br>Myeloid<br>Hematopoiesis<br>Hematopoietic
Modeling Methods	Network Model<br>Boolean Network<br>Qualitative Network<br>Logic Model<br>Logical Model<br>Boolean Model<br>Qualitative Model<br>Network Models<br>Boolean Networks<br>Qualitative Networks<br>Logic Models<br>Logical Models<br>Network Modeling<br>Logic Modeling<br>Logical Modeling<br>Boolean Modeling<br>Qualitative Modeling

Table S2: Summary of the identified AML-related logic models.

DOI	Model Type	Topic	Purpose	Knowledge Source	Verification	Availability
10.1073/pnas.1610622114	Boolean network	myeloid and lymphoid development	Understand cellular behavior	ChIP-seq data of myeloid and lymphoid cells from GEO + Literature review	In silico	SBML and GINsim model available
10.3390/jpm11030193	Probabilistic Boolean Model	FLT3-mutant AML	Drug response	RNA-seq data of MV4-11 cells under 6 drug treatment conditions including quizartinib and dexamethasone + databases interactions (SIGNOR, TRRUST, RegNetwork)	NA	Txt files of Probabilistic and deterministic Boolean networks are available
10.1016/j.csbj.2022.10.040	Boolean network	HSC aging	Understand cellular behavior	Single cell RNA-seq analysis of mouse HSC and HSPC + Previous model (PMID: 21853041)	In silico	Full description in Fig.
10.1158/0008-5472.CAN-16-1578	Multi-valued logic model	AML	Drug response	Reverse-phase protein array (RPPA) of AML cell lines + literature review	in silico & in vitro	Full description in text.
10.3390/jpm11020117	Boolean network	AML	Clinical outcome prediction	Literature review	In silico	Full description in text.

10.1186/s12859-018-2034-4	Boolean network	AML	Clinical outcome prediction	Proteomics dataset of the AML DREAM 9 challenge (RPPA of patients) + KEGG PKN	In silico	Full description in Fig.
10.1038/s41467-022-33189-w	Multi-valued logic model	AML	Clinical outcome prediction	Literature review	In silico	Full description in text and JSON file of the model.
10.1371/journal.pone.0022649	Boolean network	Myeloid differentiation	Understand cellular behavior	Literature review	In silico	Full description in text.
10.1038/srep08190	Multi-valued logic model	CML	Drug response	Literature review	In silico	Only Figs. Rules are not clear.
10.1186/1471-2105-15-S7-S7	Boolean network	Myeloid differentiation	Understand cellular behavior	Microarray data from 3 datasets of myeloid blood cell lineage at ArrayExpress	NA	Could not find the model in the article (only a brief description)
10.1016/j.isci.2022.104951	Probabilistic Boolean network	Transdifferentiation from progenitor B cell to monocytic	Understand cellular behavior	Single-cell RNA-seq of bone marrow aspirates of healthy donors	in silico	Could not find the model in the article
10.1016/j.csbj.2021.09.012	Boolean network	HSC aging	Understand cellular behavior	Single-cell RNA sequencing data of isolated peripheral blood long-term HSCs from human	NA	Model in GitHub codes.
10.1007/978-1-4939-9224-9_11	Boolean network	HSC differentiation	Understand cellular behavior	Single-cell qRT-PCR data from hematopoietic stem and progenitor cells of mouse	NA	NA
10.1073/pnas.1610609114	Boolean network	HSC differentiation	Understand cellular behavior	Single-cell qRT-PCR data from hematopoietic stem and progenitor cells of mouse	NA	Full description in text.
10.1093/bioinformatics/btx736	Boolean network	Granulocyte-monocyte precursor-derived cells	Understand cellular behavior	Literature review	in silico	SBML files and codes are available at GitHub. The model is publicly available at Cell Collective.
10.1007/978-1-0716-2277-3_12	Boolean network	HSC homeostatic maintenance	Understand cellular behavior	Literature review	NA	NA
10.3389/fphys.2020.00848	Boolean network	HSC homeostatic maintenance	Understand cellular behavior	Literature review	in silico	Full description in text.
10.1093/bioinformatics/btt243	Boolean network	Early blood stem cell development	Understand cellular behavior	Literature review	in silico & in vitro	Full description in text.
10.7554/eLife.90532.2	Boolean network	AML	Drug response	SIGNOR + phosphorylation levels of sentinel proteins under 16 differen perturbation conditions in TKIs sensitive and resistant cells	in silico & in vitro	Model file and codes are available at GitHub.

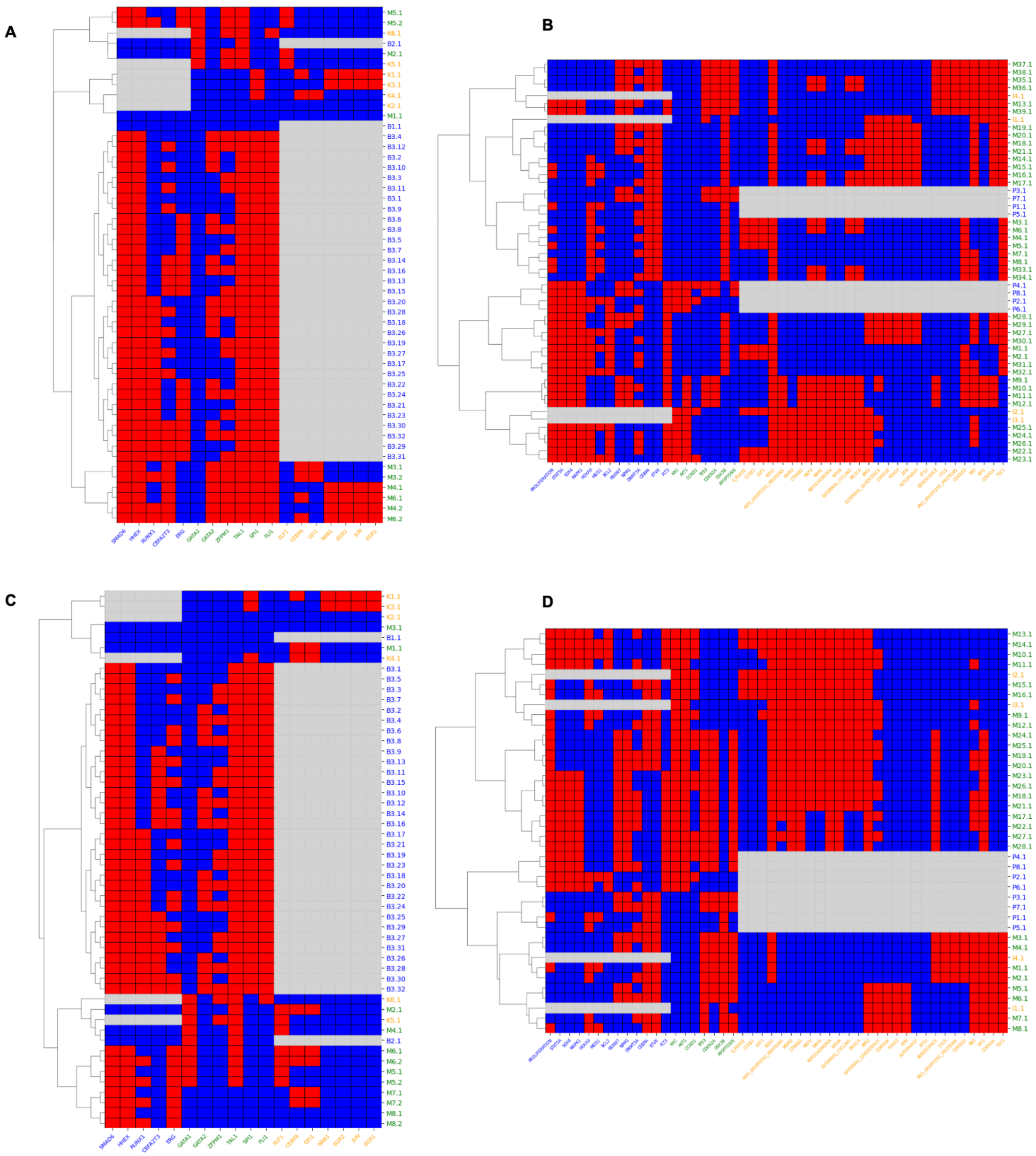

Fig S1: Steady states pattern of the merged models.

(A) Steady states pattern of the Bonzanni - Krumsiek merged model pair using the ‘OR’ approach. (B) Steady states pattern of the Palma - Ikonomi merged model pair using the ‘OR’ approach. (C) Steady states pattern of the Bonzanni - Krumsiek merged model pair using the ‘AND’ approach. (D) Steady states pattern of the Palma - Ikonomi merged model pair using the ‘AND’ approach. Steady states of both the individual models and merged models are calculated using the asynchronous approach and compared in the heatmap. The color in the heatmap indicates that a gene is ON (Blue), OFF(Red), or that the gene is not included in the model (Grey). For the axis labels, blue and orange indicate genes (x axis) or states (y axis) of the individual models, and green indicates genes shared by both models (x axis) or states of the merged model (y axis).

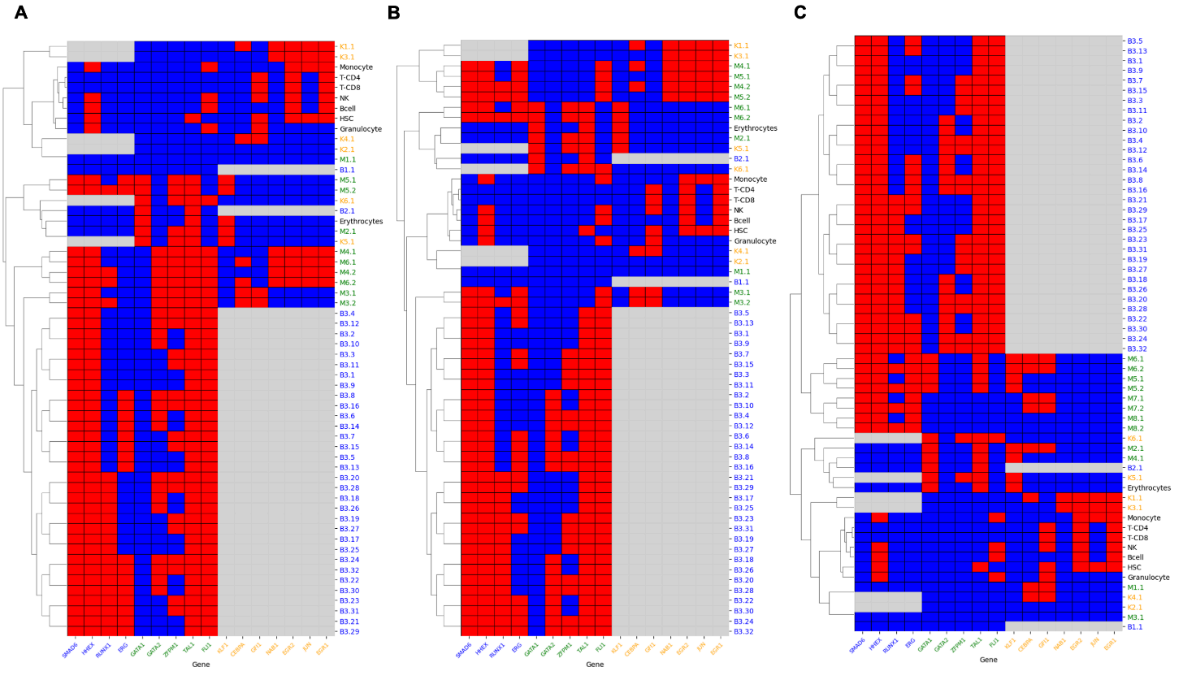

Fig S2: Compare steady states of the merged models with gene expression profiles of blood cells.

(A) Steady states of the Bonzanni - Krumsiek merged model pair using the ‘OR’ approach are clustered with gene expression of blood cells from Chambers et al. Color scales are similar as Figure S1, except that the black legends in the y-axis represent experimental data of each blood cell. (B) Steady states of the Bonzanni - Krumsiek merged model pair using the ‘Inhibitor wins’ approach (C) Steady states pattern of the Bonzanni - Krumsiek merged model pair using the ‘AND’ approach.

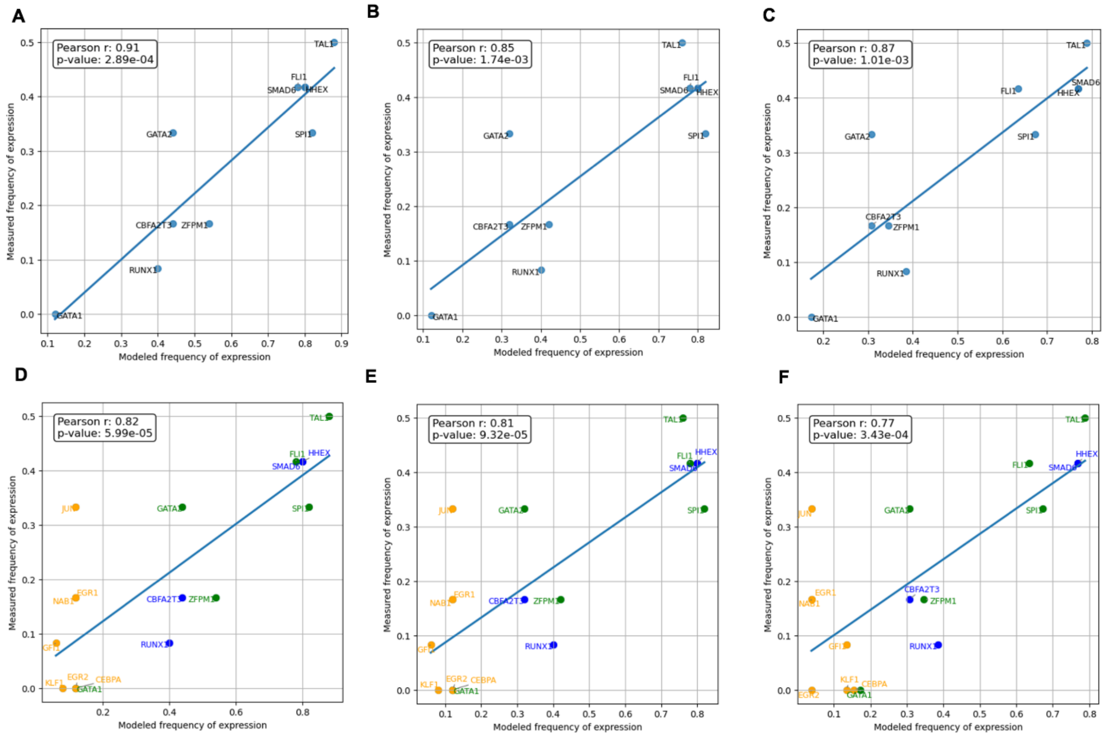

Fig S3: Expanding gene behavior modeling through merged models.

(A - C) Correlation of averaged gene expression from the 12 single-cell profiles from a previous study (Ramos et al., 2006) to the average gene expression from the modeled steady states for each of the 10 genes in the Bonzanni et al. model using the merged model of ‘OR’, ‘Inhibitor wins’ and ‘AND’ approach, respectively. (D - F) Correlation of the gene expression data with the average gene expression from the steady states of 18 genes using the merged model of ‘OR’, ‘Inhibitor wins’ and ‘AND’ approach, respectively. 8 additional genes not covered in the Bonzanni et al. model are colored in orange, together with genes covered in the Bonzanni et al. model only (blue) and shared by both models (green). Scatterplot of the ‘AND’ model are shown, for other merged models results, see Fig S3 and S4.

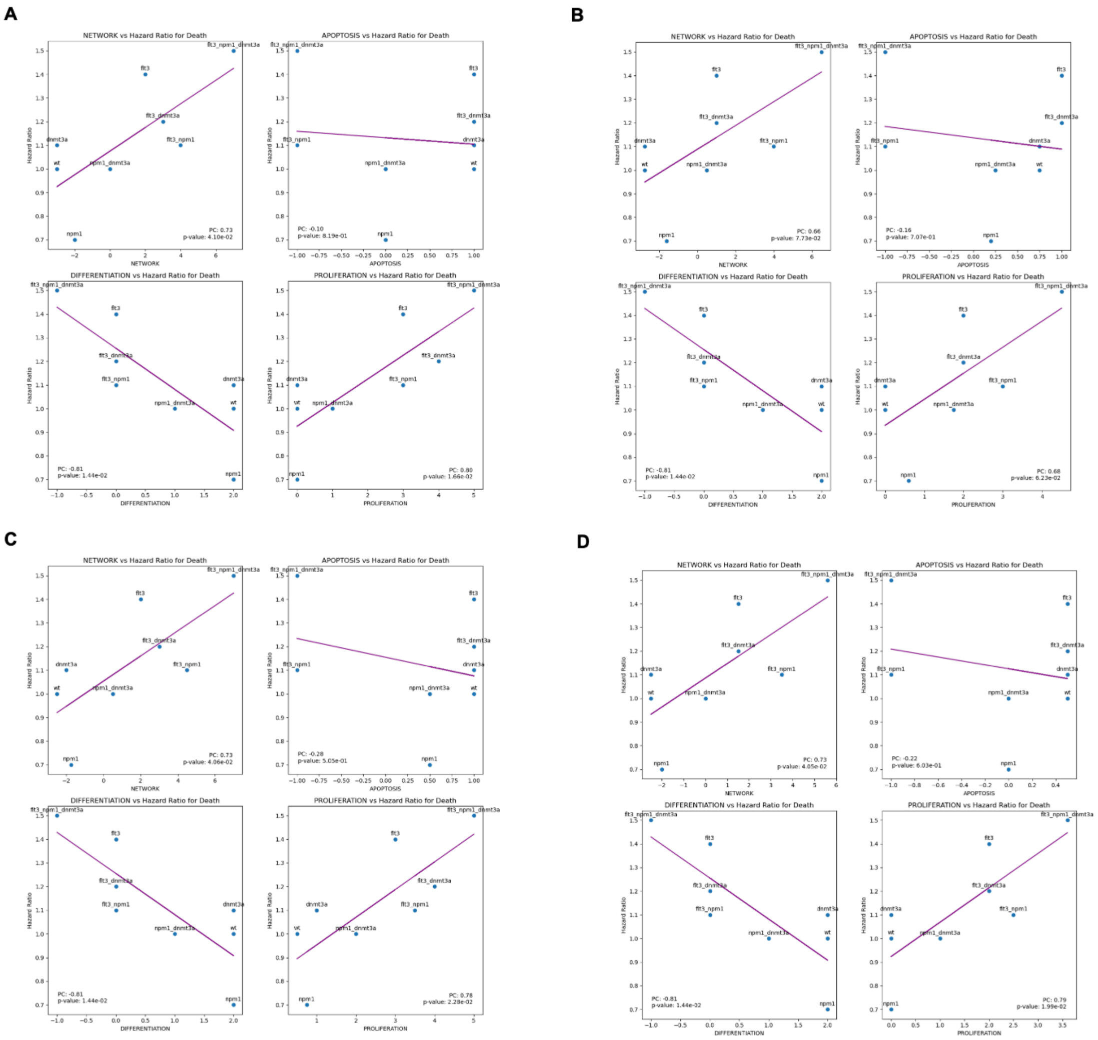

Fig S4: Reproduce the Palma results - correlation with hazard ratio for death.

As described by Palma et al., we evaluated whether the merged Palma-Ikonomi model could be used to infer clinical outcomes of patients with specific mutations. (A) Scatterplot compares the mutation-specific hazard ratios from a clinical study (Papaemmanuil et al., NEJM 2016) with the output of the Palma model. (B - D) Scatterplot compares the mutation-specific hazard ratios from a clinical study (Papaemmanuil et al., NEJM 2016) with the output of the merged model using ‘OR’, ‘Inhibitor wins’ and ‘AND’ approach, respectively. In each panel, the top-left graph shows the correlation between the network score and hazard ratio for death; other graphs show the phenotype scores of apoptosis, differentiation and proliferation, respectively.

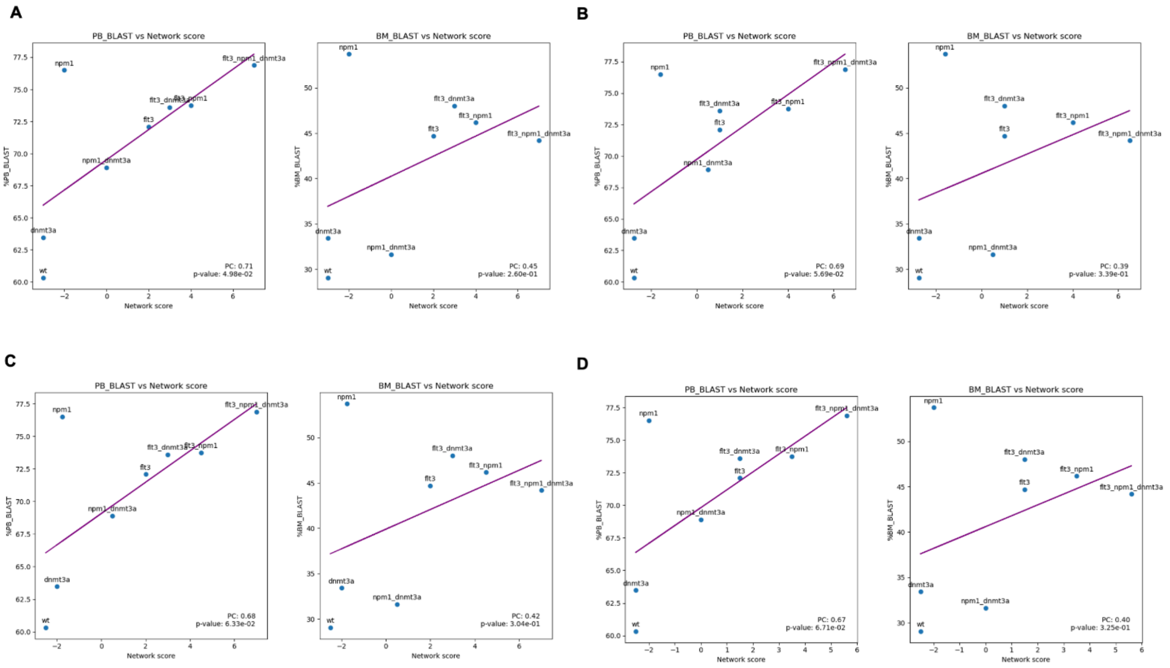

Fig S5: Reproduce the Palma results - correlation with blast percentage.

Palma et al. had tested the prognostic power of their model on patient data from the TCGA-LAML dataset. (A) Scatterplots compare the peripheral blood (PB, on the left) and bone marrow (BM, on the right) blast percentages of patients with the network scores generated by the Palma model. (B - D) Scatterplots compare the peripheral blood and bone marrow blast percentages of patients with the network scores generated by the merged models using 'OR', 'Inhibitor wins' and 'AND' approach, respectively.

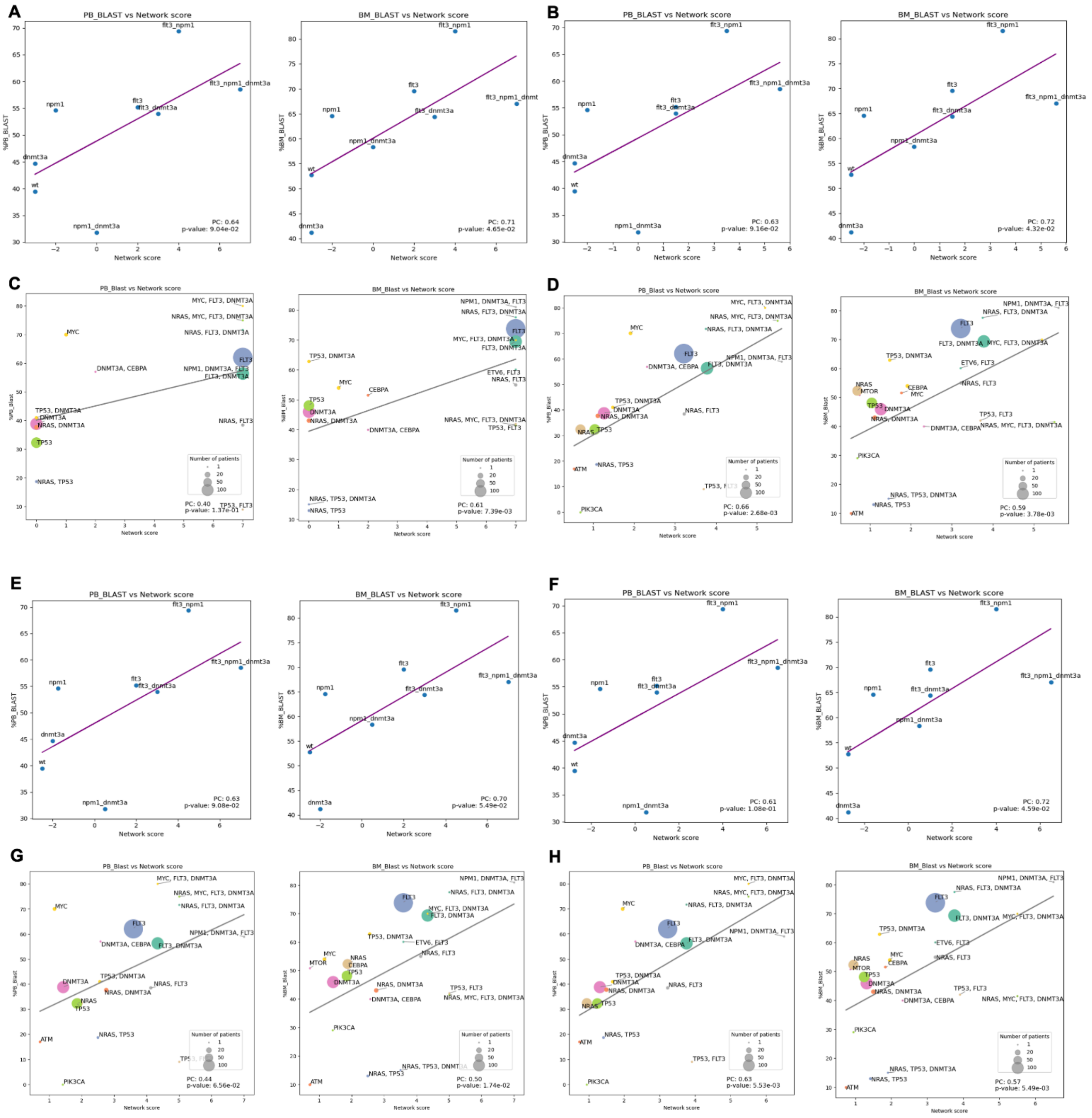

Fig S6: Expanding clinical outcome modeling through merged models.

Beat AML, a larger AML dataset, was used to evaluate the models' performance on a more diverse patient population. (A) The correlation between blast percentage and network scores derived from the model for the Palma et al. model. The left plot in each panel displays results for PB_blast, and the right panel for BM_blast. (B, E, F) The correlation between blast percentage and network scores derived from the merged models using 'OR', 'Inhibitor wins' and 'AND' approach, respectively. (C) Correlation between the average blast percentage and network scores derived from the Palma et al. model for different mutation status on all available genes in the Palma et al. model. Size of node indicates number of patients for each mutation profile. (D, G, H) Correlation between the average blast percentage and network scores derived from the merged model for different mutation status on all available genes using the merged models of 'OR', 'Inhibitor wins' and 'AND' approach, respectively.

A

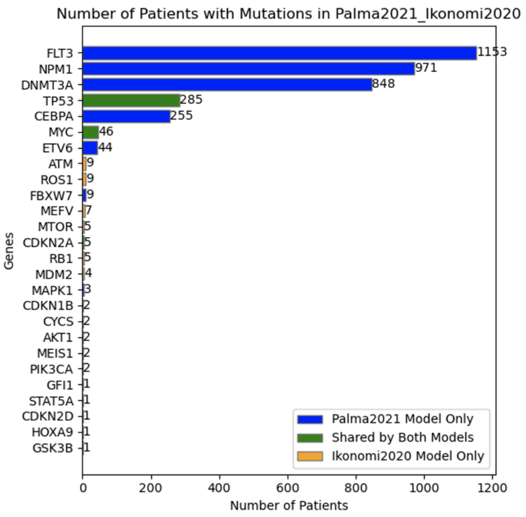

B

	cBioPortal			AMLSG		
	Palma	Ikonomi	Merged	Palma	Ikonomi	Merged
AKT1	2	2	**2**			
ATM	0	9	**9**			
CDKN1B	0	2	**2**			
CDKN2A	3	3	**3**	2	2	**2**
CDKN2D	0	1	**1**			
CEBPA	117	0	**117**	138	0	**138**
CYCS	0	2	**2**			
DNMT3A	486	0	**486**	362	0	**362**
ETV6	23	0	**23**	21	0	**21**
FBXW7				9	0	**9**
FLT3	638	0	**638**	515	0	**515**
GFI1	0	1	**1**			
GSK3B	1	1	**1**			
HOXA9	1	0	**1**			
MAPK1	3	0	**3**			
MDM2	0	4	**4**			
MEFV	0	7	**7**			
MEIS1	2	0	**2**			
MTOR	0	5	**5**			
MYC	18	18	**18**	28	28	**28**
NPM1	531	0	**531**	440	0	**440**
PIK3CA	0	2	**2**			
RB1				0	5	**5**
ROS1	0	9	**9**			
STAT5A	1	0	**1**			
TP53	184	184	**184**	101	101	**101**
Total	2010	250	**2052**	1616	136	**1621**

C

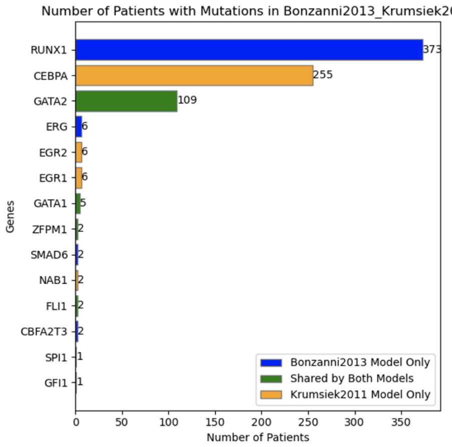

D

	cBioPortal			AMLSG		
	Bonzanni	Krumsiek	Merged	Bonzanni	Krumsiek	Merged
CBFA2T3	2	0	**2**			
CEBPA	0	117	**117**	0	138	**138**
EGR1	0	6	**6**			
EGR2	0	6	**6**			
ERG	6	0	**6**			
FLI1	2	2	**2**			
GATA1	5	5	**5**			
GATA2	72	72	**72**	37	37	**37**
GFI1	0	1	**1**			
NAB1	0	2	**2**			
RUNX1	236	0	**236**	137	0	**137**
SMAD6	2	0	**2**			
SPI1	1	1	**1**			
Total	328	214	**460**	174	175	**312**

Fig S7: Number of patients from three AML datasets whose mutations are covered in the merged model.

(A) Coverage of the patients with mutations in genes that are modeled in the Palma-Ikonomi model pair using AML datasets: cBioPortal and AMLSG. Each dataset provides gene mutation information of AML patients through targeted sequencing on somatic mutations associated with myeloid and cancer driver genes. Specifically, cBioPortal integrates patient information from 6 large AML studies, a total of 2866 patients, and AMLSG covers 1540 patients. Number of patients with each gene variant are counted and summed to reflect the overall coverage. Blue and yellow indicate that the corresponding genes are covered in the first/second model respectively, while green bars show the overlapping genes shared by both models. (B) Coverage of the patients with mutations in genes that are modeled in the Bonzanni-Krumsiek model pair using AML datasets: cBioPortal and AMLSG. (C) Breakdowns of the number of patients covered in each dataset of the Palma-Ikonomi model pair. (D) Breakdowns of the number of patients covered in each dataset of the Bonzanni-Krumsiek model pair.

.
